# Investigation into Photolithography Process of FPCB with 18 µm Line Pitch

**DOI:** 10.3390/mi14051020

**Published:** 2023-05-10

**Authors:** Ke Sun, Gai Wu, Kang Liang, Bin Sun, Jian Wang

**Affiliations:** 1 The Institute of Technological Sciences, Wuhan University, Wuhan 430072, China; sunke1720@whu.edu.cn; 2 School of Power and Mechanical Engineering, Wuhan University, Wuhan 430072, China; 3 Jiangsu Leader-Tech Semiconductor Co., Ltd., Pizhou 221300, China; sunbin@leader-techcn.com (B.S.); wangjian@leader-techcn.com (J.W.)

**Keywords:** lithography simulation, FPCB, FDTD

## Abstract

Due to the widespread application of flexible printed circuit boards (FPCBs), attention is increasing being paid to photolithography simulation with the continuous development of ultraviolet (UV) photolithography manufacturing. This study investigates the exposure process of an FPCB with an 18 µm line pitch. Using the finite difference time domain method, the light intensity distribution was calculated to predict the profiles of the developed photoresist. Moreover, the parameters of incident light intensity, air gap, and types of media that significantly influence the profile quality were studied. Using the process parameters obtained by photolithography simulation, FPCB samples with an 18 µm line pitch were successfully prepared. The results show that a higher incident light intensity and a smaller air gap result in a larger photoresisst profile. Better profile quality was obtained when water was used as the medium. The reliability of the simulation model was validated by comparing the profiles of the developed photoresist via four experimental samples.

## 1. Introduction

Bearing the benefits of good portability [1], light weight, small size, and excellent bending performance, flexible printed circuit boards (FPCBs) are widely used in mobile communication equipments, flexible wearable devices [2], and automotive electronic products [3]. With increasing demand in the FPCB market, the photolithography process [4] has been a crucial driving force for achieving a narrower FPCB line pitch. In the process of ultraviolet (UV) lithography [5,6], patterns are constructed in a light-sensitive material [7] (photoresist) using ultraviolet light. During the exposure process [8], part of the light is absorbed by the photoresist [9], which generates photoacids [10] that change its solubility in the developer. Obtaining the desired size of the pattern requires controlling the photoacid distribution related to the light intensity distribution [11]. The expensive equipments and complicated process steps of the lithography process are time-consuming and costly. Lithography simulation technology can be used to study the propagation process of light in the photoresist. It has become an efficient means for analyzing and optimizing the manufacturing process and it efficiently reduces time and cost.

Currently, some investigations are being carried out into lithography simulation. Based on the scalar diffraction theory, Bourdillon et al. [12] used the Fresnel diffraction model to simulate 1 nm-proximity X-ray lithography. They demonstrated the influences of the gap width, spectral bandwidth, outriggers, T junctions, blur, etc. Tian et al. [13] simulated deep ultraviolet (DUV) photolithography with regard to the SU-8 photoresist using the modified Fresnel diffraction model, which can achieve a higher precision simulation than the Fresnel diffraction model. Different from the traditional Fresnel diffraction model, the modified Fresnel diffraction model divides the mask area into countless small squares to study the heterogeneity of diffraction in different directions. If the squares are small enough, the results can be very accurate. Zhou et al. [14] proposed a comprehensive aerial image model using Fresnel diffraction to calculate the three-dimensional (3D) inclined/vertical UV light intensity distribution in SU-8. Meanwhile, Geng et al. [15] constructed a high precision photolithography simulation of the thick SU-8 photoresist using the waveguide method (WG) in two dimensions and predicted the profiles of the photoresist based on the calculated light intensity distribution in SU-8. Koyama et al. [16] simulated the UV curing process of the photoresist via nanoimprint photolithography utilizing the molecular mechanics method. Majumder et al. [17] designed a full electromagnetic wave solution model using the finite element method to simulate the photochemical processes involved in absorbance modulation optical lithography. Kerim et al. [18] simulated electron beam lithography in relation to curved and inclined surfaces using a graphical-processing-unit-accelerated 3D Monte Carlo simulation based on first-principle scattering models. Liu et al. [19] developed a fast model for simulating the mask diffraction spectrum of extreme ultraviolet photolithography by combining an improved thin mask model and an equivalent layer method to simulate the mask diffraction for 22 nm space features. However, none of these studies have investigated 3D photolithography simulations based on the finite difference time domain (FDTD) method. Moreover, at present, many companies have achieved the manufacturing of FPCBs with a 20 μm line pitch, such as the LG company in South Korea, Chipbond Technology Co., Ltd., Taiwan, China and FLEXCEED Co., Ltd., Naka-shi, Japan [2]. The breakthrough in the production process of an FPCB with a 20 μm line pitch plays an extremely important driving role in the development of the flexible electronics field. The manufacturing of FPCBs with an 18 μm line pitch is the next important step in the development of the FPCBs. The production technology of an FPCB with an 18 μm line pitch or narrower line pitch is still not clear.

In this study, the exposure process of FPCBs with an 18 µm line pitch is studied. A 3D optical model of the FPCB is established and simulated using the FDTD method to obtained the profiles of the developed photoresist structures. The simulated profiles of the developed photoresist predicted via the light intensity distribution in the photoresist are compared with the experimental results for model validation purposes.

## 2. Materials and Methods

### 2.1. Governing Equations

**Figure 1:**
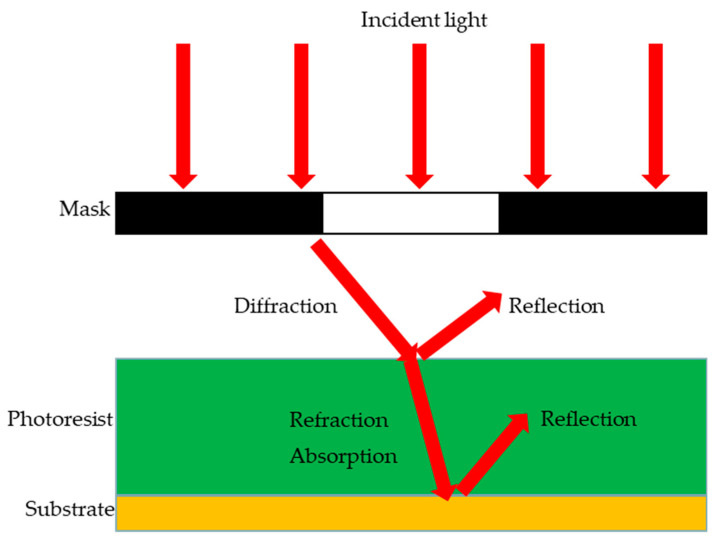
Schematic of the exposure process.

Figure 1 shows the schematic of the exposure process. The incident light is assumed to be a beam of uniform and parallel light perpendicular to the mask surface. Diffraction occurs when the light passes through the mask. When the light passes through the photoresist surface, part of the light is refracted into the photoresist. The other part is reflected back into the air via the photoresist surface. The light entering the photoresist is partly absorbed by the photoresist to form photoacid. The light not absorbed by the photoresist is transmitted through the photoresist to the substrate, which is reflected back into the photoresist and superimposed with the incident light.

The main lithography simulation methods include the Fresnel diffraction model, FEM, WG, and FDTD. The Fresnel diffraction model considers the electric and magnetic field components in Maxwell’s equations as scalars, without considering the actual coupling between the electric and magnetic vectors. It greatly simplifies the diffraction process, reduces computational complexity and improves computational speed, but the calculation is not accurate enough. Different from the the Fresnel diffraction model, FEM, WG and FDTD are based on the rigorous electromagnetic field theory, considering electromagnetic field components as vectors to achieve the accurate simulation of diffraction processes. The FEM has very high computational accuracy but requires a large amount of computing resources and time. The WG method has a shorter computing time but lower calculation accuracy than the FDTD method. The FDTD method has the advantage of high computational accuracy and wide applicability and the calculation time can be reduced via parallel computing. Therefore, in order to obtain appropriate simulation accuracy and computation time, we finally chosed the FDTD method to calculate the light intensity distribution. Maxwell’s equations are the core equations that explain the propagation process of the electromagnetic waves in nonmagnetic materials. In modern notation, Maxwell’s equations are presented as follows:

(1) $$\frac{\partial D}{\partial t}=\nabla \times H-J$$

(2) $$-\frac{\partial B}{\partial t}=\nabla \times E$$

where ***D*** and ***H*** denote the electric displacement and the magnetic field vector, respectively; *t* is time; ***J*** is the electric charge current density; ***B*** is the magnetic flux density; ***E*** is the electric field intensity; and ∇ is the gradient differential operator.

The constitutive relationship is a necessary condition for supplementing Maxwell’s equations and characterizing the material parameters. The constitutive relationships beteeen the isotropic linear materials are as follows:

(3) D=εE

(4) B=μH

(5) J=σE

where *ε* is the dielectric constant of the medium, *μ* is the magnetic permeability, and *σ* is the electrical conductivity.

In a rectangular coordinate system, Equations (1) and (2) can be transformed into the following form:

(6) $$\frac{\partial H_{z}}{\partial y}-\frac{\partial H_{y}}{\partial z}=\varepsilon \frac{\partial E_{x}}{\partial t}+\sigma E_{x}$$

(7) $$\frac{\partial H_{x}}{\partial z}-\frac{\partial H_{z}}{\partial x}=\varepsilon \frac{\partial E_{y}}{\partial t}+\sigma E_{y}$$

(8) $$\frac{\partial H_{y}}{\partial x}-\frac{\partial H_{x}}{\partial y}=\varepsilon \frac{\partial E_{z}}{\partial t}+\sigma E_{z}$$

(9) $$\frac{\partial E_{z}}{\partial y}-\frac{\partial E_{y}}{\partial z}=-\mu \frac{\partial H_{x}}{\partial t}$$

(10) $$\frac{\partial E_{x}}{\partial z}-\frac{\partial E_{z}}{\partial x}=\mu \frac{\partial H_{y}}{\partial t}$$

(11) $$\frac{\partial E_{y}}{\partial x}-\frac{\partial E_{x}}{\partial y}=\mu \frac{\partial H_{z}}{\partial t}$$

**Figure 2:**
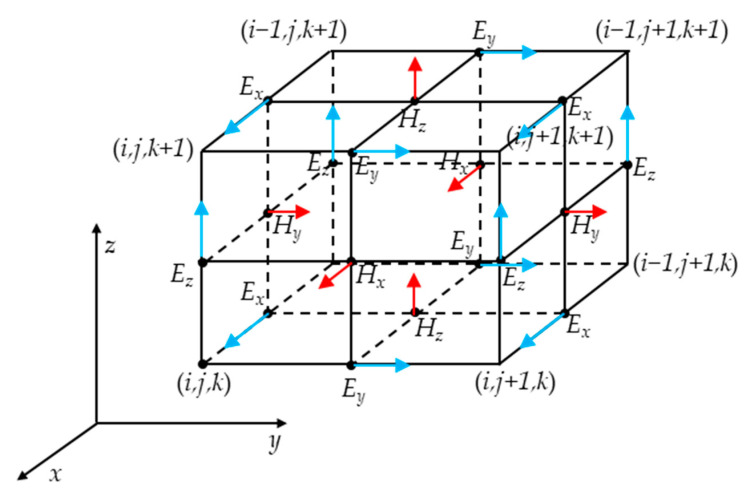
Positions and directions of various field components.

Using the central difference formula to replace the first order partial derivative, the three-dimensional electric and magnetic fields were sampled and calculated at discrete positions in time and space. *Fq* (*i*, *j*, *k*) was assumed to be the discrete value of a component of the electric field *E* or the magnetic field *H* when the time was *t* and the coordinate was (*i*, *j*, *k*) in the rectangular coordinate system and *q* = *x*, *y*, *z*. In space, different components with the same index (*i*, *j*, *k*) can form a specific rectangular Yee cell. Figure 2 shows the actual position of each point in the Yee cell. The FDTD method was used to perform instantaneous sampling and to calculate of electric and magnetic field components at different discrete times. The electric field component corresponds to time ∆*t*, 2 ∆*t*, 3 ∆*t*, …, *n* ∆*t*, while the magnetic field component corresponds to time 1.5 ∆*t*, 2.5 ∆*t*, 3.5 ∆*t*, …, (*n* + 0.5) ∆*t*, with the offset always being 0.5 ∆*t*. In the subsequent processing of the calculation results, it was necessary to unify the electric and magnetic field components to the same time.

Figure 2 shows that the electric field component nodes are located at the center of the edge of the cell and the vector direction is parallel to their respective edges, while the magnetic field component nodes are located at the center of each surface and the direction is perpendicular to the corresponding surface. This also means that each electric field component node is surrounded by four magnetic field component nodes, simulating Ampere’s law, while each magnetic field component node is surrounded by four electric field components, simulating Faraday’s law.

The central difference equation is as follows:

(12) $$\frac{\partial F_{x}{}^{t}(i,j,k)}{\partial x}\left|{}_{x=i\Delta x}\right.=\frac{F_{x}{}^{t}(i+0.5,j,k)-F_{x}{}^{t}(i-0.5,j,k)}{\Delta x}$$

(13) $$\frac{\partial F_{q}{}^{t}(i,j,k)}{\partial x}\left|{}_{t=n\Delta t}\right.=\frac{F_{q}{}^{t+0.5}(i,j,k)-F_{q}{}^{t-0.5}(i,j,k)}{\Delta t}$$

For Equation (6), we have

(14) $$\frac{E_{x}^{n+1}(i,j,k)-E_{x}^{n}(i,j,k)}{\Delta t}+\frac{\sigma_{x}(i,j,k)}{\varepsilon_{x}(i,j,k)}E_{x}^{n+0.5}(i,j,k)=\frac{1}{\varepsilon_{x}(i,j,k)}(\frac{H_{z}^{n+0.5}(i,j,k)-H_{z}^{n+0.5}(i,j-1,k)}{\Delta y}-\frac{H_{y}^{n+0.5}(i,j,k)-H_{y}^{n+0.5}(i,j,k-1)}{\Delta z})$$

The equations corresponding to (7) and (8), respectively, can be similarly constructed.

For Equation (9), we have

(15) $$H_{x}^{n+0.5}(i,j,k)=H_{x}^{n-0.5}(i,j,k)-\frac{\Delta t}{\mu_{x}(i,j,k)}(\frac{E_{z}^{n}(i,j+1,k)-E_{z}^{n}(i,j,k)}{\Delta y}-\frac{E_{y}^{n}(i,j,k+1)-E_{y}^{n}(i,j,k)}{\Delta z})$$

The equations corresponding to (10) and (11), respectively, can be similarly constructed.

The instantaneous value at the intermediate time is assumed to be the average value:

(16) $$F_{q}^{n+0.5}(i,j,k)=0.5\times (F_{q}^{n+1}(i,j,k)+F_{q}^{n}(i,j,k))$$

For Equation (14), we have

(17) $$E_{x}^{n+1}(i,j,k)=M_{x}(i,j,k)E_{x}^{n}(i,j,k)+N_{x}(i,j,k)(\frac{H_{z}^{n+0.5}(i,j,k)-H_{z}^{n+0.5}(i,j-1,k)}{\Delta y}-\frac{H_{y}^{n+0.5}(i,j,k)-H_{y}^{n+0.5}(i,j,k-1)}{\Delta z})$$

(18) $$M_{x}(i,j,k)=\frac{2\varepsilon_{x}(i,j,k)-\Delta t\sigma_{x}(i,j,k)}{2\varepsilon_{x}(i,j,k)-\Delta t\sigma_{x}(i,j,k)}$$

(19) $$N_{x}(i,j,k)=\frac{2\Delta t}{2\varepsilon_{x}(i,j,k)+\Delta t\sigma_{x}(i,j,k)}$$

Equations (15) and (17) are time domain update equations for electric and magnetic field components, respectively.

According to the principle of field strength superposition, the relationship between power density and electric field strength during light propagation is as follows:

(20) $$P=\frac{cn\varepsilon_{0}}{2}E^{2}$$

where *P* is the power density, *c* is the light speed, and *n* is the refractive index.

### 2.2. Simulation Model

**Figure 3:**
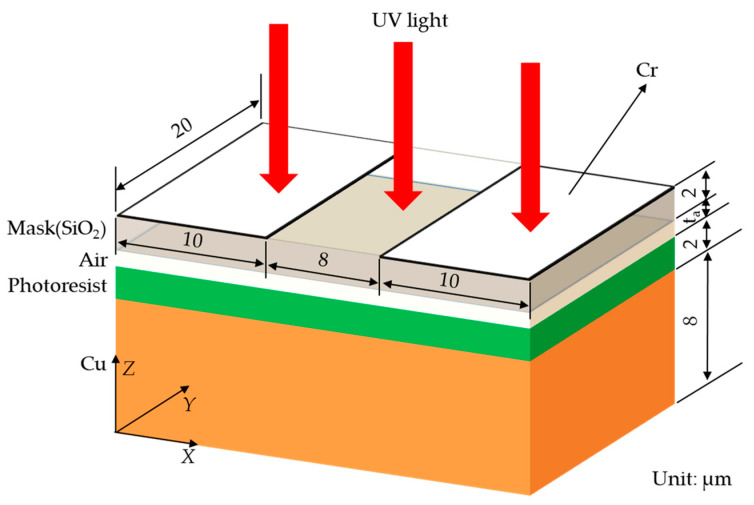
Geometric model of an FPCB circuit with an 18 µm line pitch.

Figure 3 shows the geometric model of an FPCB circuit with an 18 µm line pitch, which selected a period region in the mask pattern as the simulation object. The spacing between the neighbor Cr layers was 8 µm. The Cr layer thickness was 0.1 µm. The thickness of the mask (SiO_2_) was set to 2 µm. An air gap (*t_a_*) is defined as the distance between the mask and the photoresist layer. The computer used in this simulation could complete the calculation at micron level but not the actual meter level calculation. Thus, the simulation simplified the actual production conditions. Five air gaps (i.e., 2, 4, 6, 8, and 10 µm) are studied herein. The incident light was parallel, uniform, and perpendicular to the mask surface along the negative *Z* direction, and its wavelength was 365 nm. The incident light was a plane wave in this simulation. The corresponding refractive index and permittivity of the materials with a 365 nm wavelength in the model are shown in Table 1. Among them, the material parameters of SiO_2_, Cr, and copper can be found in Handbook of Optical Constants of Solids [20]. The material parameters of the photoresist were provided by Jiangsu Leader-Tech Semiconductor Co., Ltd., Pizhou, China. The data of SiO_2_, Cr, and copper is derived from experiments described by sample data model. And the parameters of photoresist is set by (*n*, *k*) material model.

In this work, all numerical simulations were performed using Lumerical 2020 R2 (ANSYS Inc., Pittsburgh, PA, USA), which is based on the FDTD method. Considering the periodicity and the symmetry of the simulation model in the *X* and *Y* directions, a plane wave source and periodic boundary conditions were added to save the computation resources and reduce the calculation time. Meanwhile, the symmetric boundary conditions in the *X* and *Y* directions were set to reduce the memory and time required for calculations. The mesh was auto non-uniform. The second level of mesh accuracy was used to complete the simulation calculations and the minimum mesh step was 0.00045 µm. The perfectly matched layer (PML) boundary condition with a steep angle in the *Z* direction is used to minimize the effects of reflections and improve the result accuracy. A refractive index monitor and field time monitor were set to check whether simulation calculations converge sufficiently to confirm the reliability of the simulation. Additionally, a 3D frequency domain-field and power monitor is added to collect electric field data from the photoresist.

## 3. Results and Discussion

### 3.1. Effects of Incident Light Intensity

**Figure 4:**
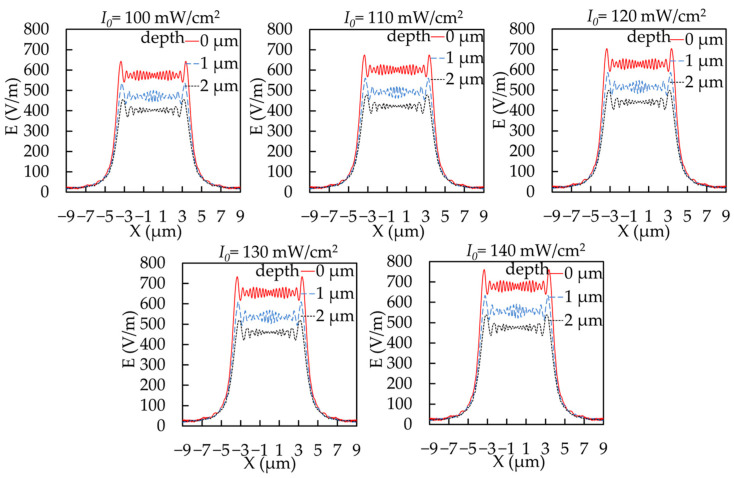
Intensity curves for the vertical exposure at different depths.

**Figure 5:**
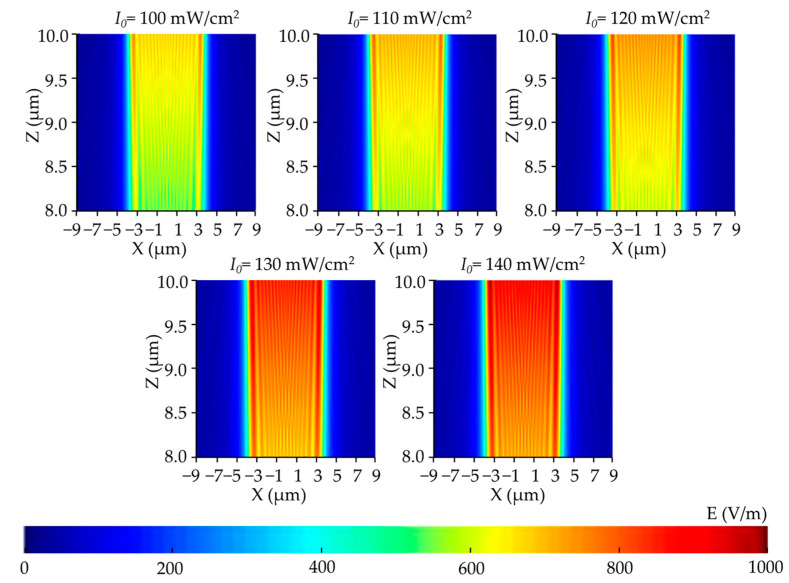
Vertical cross-section distribution of the electric field intensity in the photoresist with different incident light intensities.

**Figure 6:**
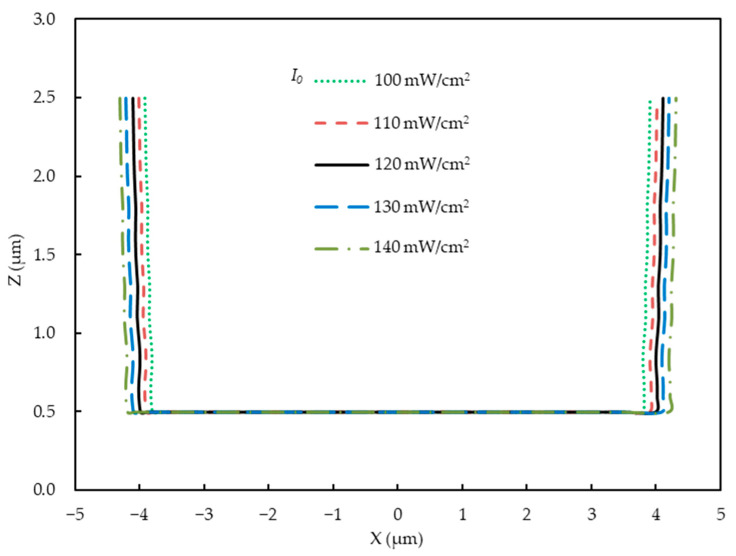
Simulated profiles of the developed photoresist with different incident light intensities.

Keeping the other parameters invariant, we focus herein on the effects of the incident light intensity. Figure 4, Figure 5 and Figure 6 present the simulation results.

Figure 4 shows the intensity curves for the vertical exposure of different depths with varying incident light intensities. Via the screening effect of the mask on light, the light intensity reached its peak at the edge of the exposure area, and fluctuated continuously around a certain value within the exposure area, as shown in Figure 4. Due to the incident light diffraction and absorption in the photoresist, its intensity was simultaneously attenuated along the radiation direction with its increasing depths. According to the traditional optics theory, Fresnel diffraction exists near the photoresist surface and gradually changes to Fraunhofer diffraction with a depth increase. Due to the small size of the transmittance region (Figure 4), the Fresnel diffraction rapidly degenerated to Fraunhofer diffraction with increasing depths, where the intensity at the same depth sharply fluctuated in the x direction. Meanwhile, Figure 4 shows that the shape of the light intensity curve under different incident light intensities was basically the same, but the average value of light intensity increased with the increase of the incident light intensity. This indicates that a change in incident light intensity does not affect the propagation characteristics of light during the exposure process, but only affects the intensity of the light field in the photoresist.

Figure 5 illustrates the corresponding contour maps. We can see from the results that the light field was mainly distributed in the exposure area of the corresponding mask, while the light intensity in the non-exposure area was extremely low and can be ignored. Evidently, the effectiveness of the mask has been well demonstrated. In Figure 5, as the incident light intensity increased from 100 to 140 mW/cm^2^, the light intensity in the exposed area increased, the exposure area shifted to the sides, the diffraction effect became more intense.

Figure 6 displays the profiles of the developed photoresist structures predicted via the light intensity distribution with different incident light intensities. The photoresist profile in Figure 6 was extracted from the light intensity distribution of the photoresist (Figure 5), which is the boundary between the exposed area and the unexposed area in the vertical section. At the same time, it was assumed that the development process was perfect, and so the profile of the exposure area extracted from the light intensity distribution can be seen as equivalent to the profile of the developed photoresist. The bottom of the profiles of the developed photoresist structures had widths of 7.78 µm, 7.88 µm, 8.01 µm, 8.13 µm, and 8.26 µm, respectively. In actual production, an 8 µm bottom width is ideal for obtaining an FPCB with an 18 µm line pitch.

With an incident light intensity of 120 mW/cm^2^, the width at the bottom of the profiles was 8.01 µm, which was close to the ideal condition. Therefore, the incident light intensity during the production process should be controlled at approximately 120 mW/cm^2^.

### 3.2. Effects of Air Gap and Types of Media

**Figure 7:**
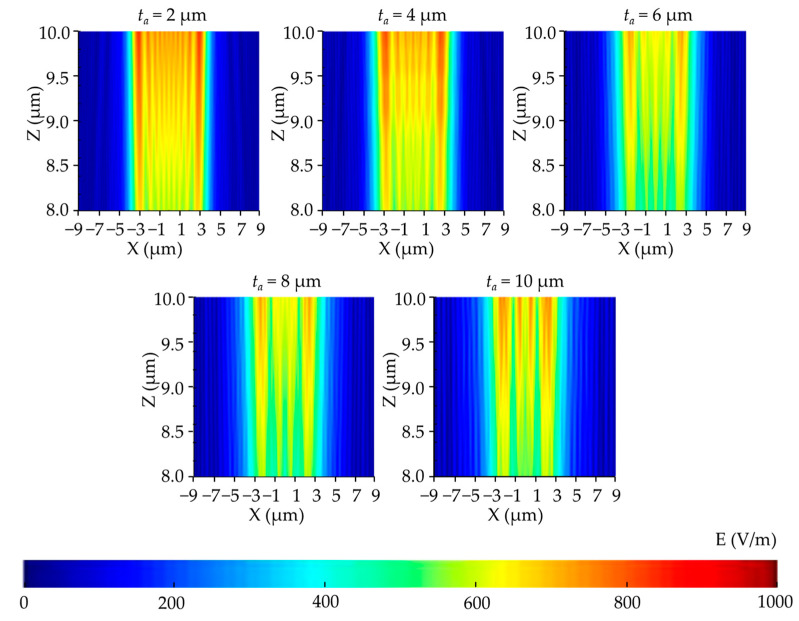
Vertical cross-section distribution of the electric field intensity in the photoresist with different air layer thicknesses.

**Figure 8:**
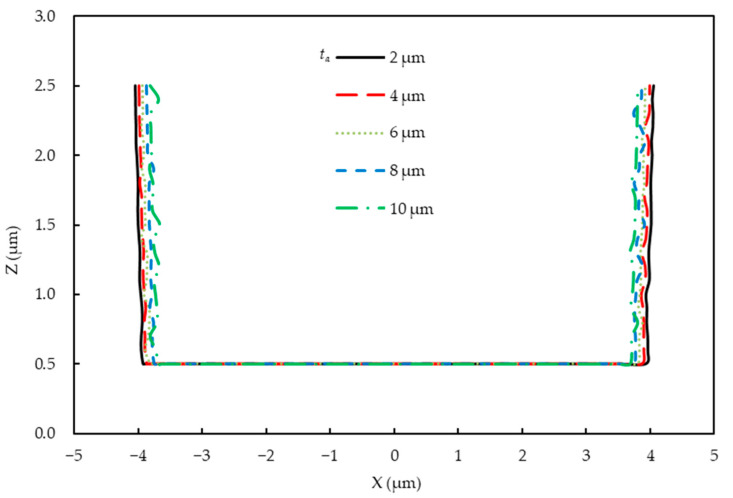
Simulated profiles of the developed photoresist with different air layer thicknesses.

A parametric study on the air gap (*t_a_*) was performed considering the influence of diffraction and absorption in air. Figure 7 shows that the light intensity distribution became non-uniform when the air gap increased. The light intensity of the photoresist reduced because when the air gap increased, more areas were generated. In these areas, Fraunhofer diffraction occured, which caused a severe intensity fluctuation. As can be seen in Figure 8, the simulated profile widths of the developed photoresist began to decrease with increasing air layer thickness because more diffraction and absorption resulted in inadequate exposure.

**Figure 9:**
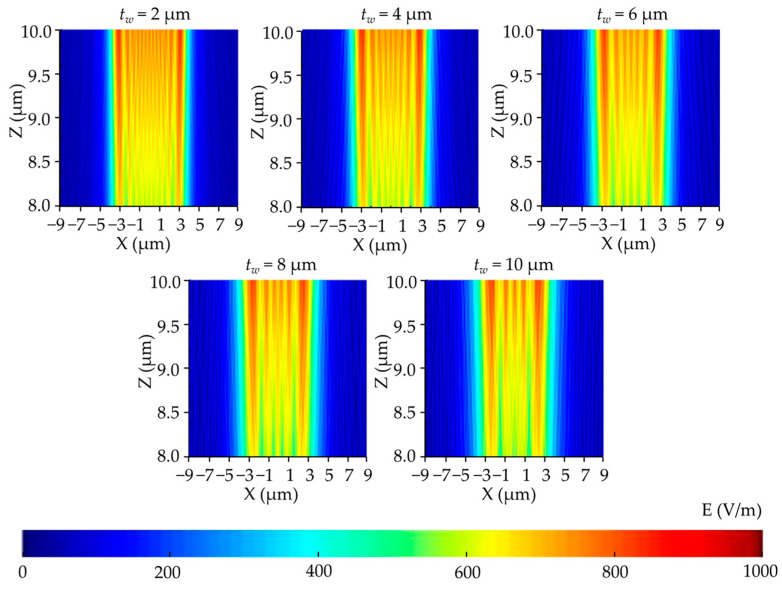
Vertical cross-section distribution of the electric field intensity in the photoresist with different water layer thicknesses.

**Figure 10:**
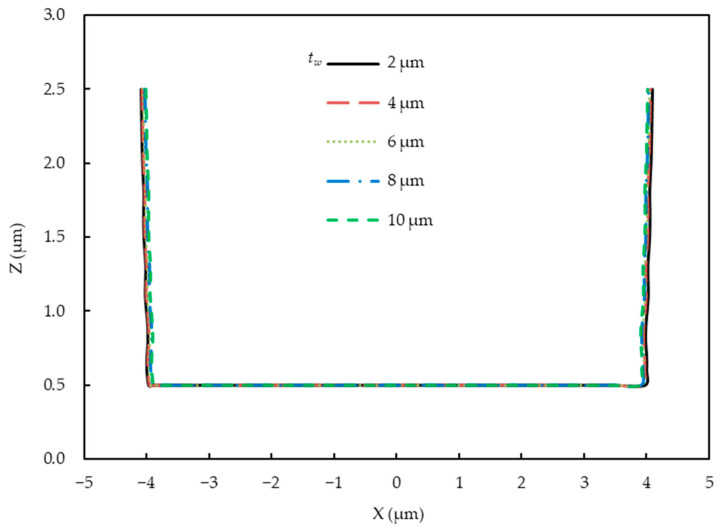
Simulated profiles of the developed photoresist with different water layer thicknesses.

The types of media between the mask and the photoresist greatly influenced the diffraction and absorption in the exposure process, thereby affecting the light intensity distribution of the photoresist. The exposure process of water being used as a medium in the field of FPCBs via simulation was explored to provide guidance for future breakthroughs in production technology. Figure 9 shows the vertical cross-section distribution of the light intensity in the photoresist with different water layer thicknesses (*t_w_*). Figure 10 depicts the developing surface displacement of the photoresist. The results show that as the water layer thickness increased from 2 to 10 µm, the light intensity decreased, but almost no significant differences were found in the simulation profiles of the FPCB compared to the condition when air was the medium between the mask and the photoresist. Water had higher refractive index and transmissivity than air when light was rapidly attenuated in water. The water layer thickness only slightly influenced the developing surface displacement of the photoresist. In other words, water will be an excellent medium for replacing air if we can solve the other problems caused by water (e.g., photoresist stability and lens cleaning).

### 3.3. Experimental Results and Analysis

**Figure 11:**
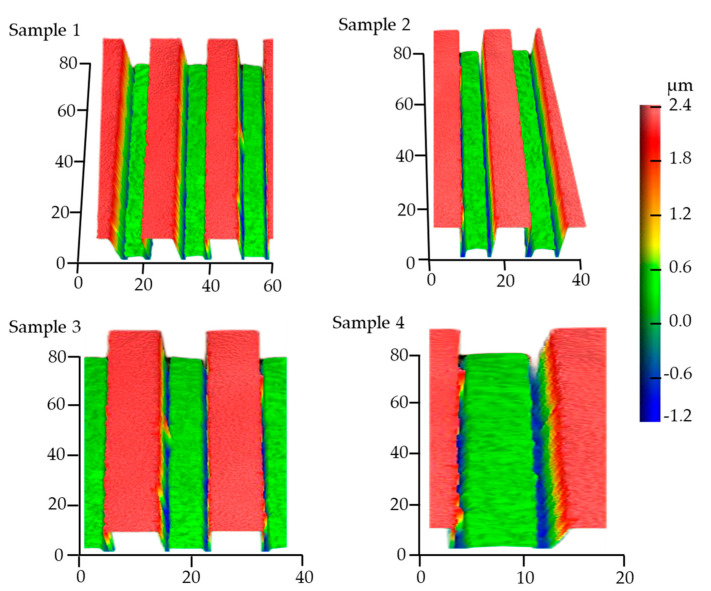
Three-dimensional profile images of four FPCB samples under the same exposure parmeters and experimental conditions.

After exposure, the photoresist was dissolved in the developer and a photoresist pattern is formed on the photoresist surface. Figure 11 shows the three-dimensional profile images of the FPCB sample (fabricated by Jiangsu Leader-Tech Semiconductor Co., Ltd.) obtained using the White Light Interferometer (Newview 9000, ZYGO, Middlefield, CT, USA) under the same exposure parameters and experimental conditions. In this experiment, the incident light intensity was 120 mW/cm^2^, and its wavelength was 365 nm. The medium between the mask and the photoresist was air. The sample was exposed via an I-line exposure machine and developed using NaOH solution.

**Figure 12:**
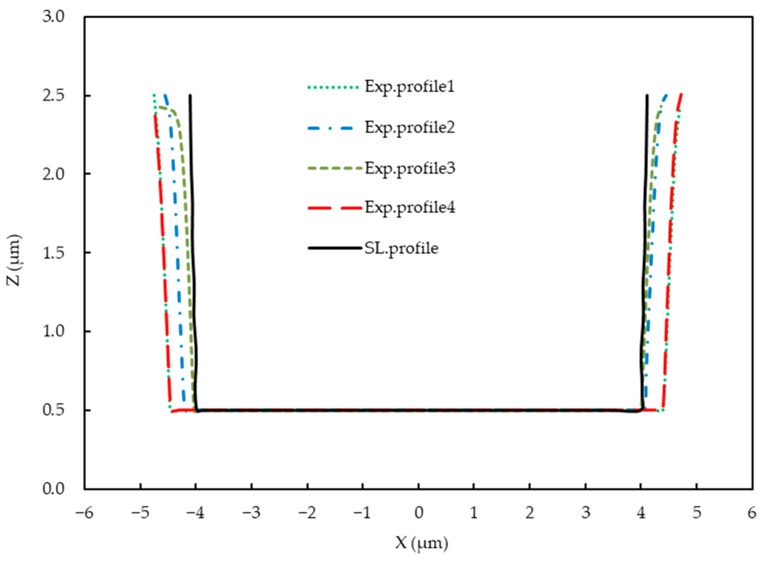
Profiles of the FPCB samples obtained via simulation and experiment.

Due to production and measurement errors, there are slight differences in the morphology of the samples. In order to reduce these errors, we produced four samples under the same parameters and production conditions. In general, the contour shapes of the samples are basically consistent and shows an isosceles trapezoid due to appropriate production parameters. Furthermore, the two-dimensional cross-section profiles of the developed photoresist were extracted from experimental results (Figure 11) and compared with the simulated profiles (Figure 12). The simulation profile (Figure 12) was obtained under the following parameters: the incident light intensity was 120 mW/cm^2^ with a wavelength of 365 nm and the air gap was 2 µm. Good agreement was found, especially in the horizontal exposure width. This validated that the proposed method can achieve accurate simulation of FPCB exposure process, and the parameters obtained from the simulation are valuable.

No significant differences were found in the maximum lateral exposure of the sidewalls, because the photoresist thickness was only 2 µm, which was not thick enough for obvious diffraction. The width at the bottom of the profiles of the developed photoresist structures mainly depended on the diffraction and absorption in the mask, air, and photoresist.

## 4. Conclusions

This paper proposed a simulation method based on the rigorous electromagnetic field theory to predict the profiles of the developed photoresist structures. A 3D simulation model of the FPCB circuit was established to study the light intensity distribution in the photoresist based on the mask, light source, and photoresist information. According to the parametric study of the quality of the profiles of the developed photoresist structures, an incident light intensity of 120 mW/cm^2^ was determined and used to expose the FPCBs. A smaller air gap can result in a larger photoresist profile. Better profile quality will be obtained when water is used as the medium. The simulation model was successfully verified by comparing the simulation and experiment results.

## Figures and Tables

**Table 1:** Material parameters in the model.

Materials	Cr	SiO_2_	Photoresist	Cu
Refractive index	1.4 + 3.26*i*	1.47	1.68 + 0.0058*i*	1.27 + 1.95*i*
Permittivity	−8.66 + 9.13*i*	2.17	2.82 + 0.019*i*	−2.21 + 4.96*i*

## Data Availability

The data presented in this study are available from the corresponding author upon request.
